# Interaction between escitalopram and risperidone

**DOI:** 10.4103/0019-5545.46081

**Published:** 2005

**Authors:** Pradeep Rao, Nishant Bhagat, Bharat Shah, Momin Bazegha

**Affiliations:** * Lecturer, Department of Psychiatry, K.J. Somaiya Medical College and Hospital, Mumbai; ** Lecturer, Department of Psychiatry, K.J. Somaiya Medical College and Hospital, Mumbai; *** Honorary Professor, Department of Psychiatry, K.J. Somaiya Medical College and Hospital, Mumbai; **** Resident, Department of Psychiatry, K.J. Somaiya Medical College and Hospital, Mumbai

**Keywords:** Escitalopram, risperidone, drug–drug interaction

## Abstract

The ever-increasing use of psychotropic drugs in clinical practice today is a blessing for the patient who can hope to achieve faster remission and possibly a higher rate of cure. However, the flip side is that the use of polypharmacy increases the potential for drug–drug interactions. Escitalopram is a novel antidepressant and there are several reports on its efficacy in the treatment of depression. Reports about its interactions with the cytochrome P450 (CYP 450) enzyme system are relatively few in number. We present a case report of a possible interaction between escitalopram and risperidone. Escitalopram has an inhibitory effect on the CYP 2D6 enzyme. Risperidone is the substrate for this enzyme. In the presence of escitalopram, there is a rise in the levels of risperidone, as evidenced by severe extrapyramidal adverse effects at a low dose of risperidone not normally known to cause extrapyramidal effects.

## INTRODUCTION

A large number of pharmacokinetic interactions take place at the level of the cytochrome P450 (CYP 450) enzyme system.^1^ Many psychotropic drugs are also metabolized by this route. In the past few years, a large number of newer psychotropic drugs have been introduced in clinical practice, bringing with them the potential for more drug interactions. The CYP 450 enzymes oxidize endogenous and exogenous compounds and render them fit for elimination. They primarily metabolize steroids and neuropeptides. Although there are more than 40 types of CYP 450 enzymes, only about 6 are clinically relevant and are responsible for more than 90% of the activity of the CYP 450 system. These are 1A2, 3A4, 2C9, 2C19, 2D6 and 2E1.^2^

An increasing pharmacopoeia and newer research into the treatment of psychiatric illnesses makes polypharmacy inevitable, especially when newer indications are being advocated for existing drugs as well. The newer psychotropic drugs (both selective serotonin reuptake inhibitors [SSRIs] and atypical antipsychotics) are extensively metabolized by the CYP 450 enzymes and are susceptible to metabolically based drug–drug interactions.^3^ We report a case of possible interaction between escitalopram and risperidone.

## THE CASE

A 19-year-old engineering college student presented with symptoms of sadness of mood, disinterest in activities and academics, inability to concentrate on studies, lack of confidence, low self-esteem, ideas of helplessness and hopelessness, and suicidal thoughts, following an unexpected poor performance in an academic presentation 2 months ago. He was diagnosed with major depressive disorder and was started on escitalopram 10 mg once a day and amitriptyline 25 mg three times a day. He was admitted in view of the severity of depression and suicidal ideation, and was also treated with electroconvulsive therapy (ECT). At the end of the course of ECT, his condition improved significantly and he was discharged on escitalopram and amitriptyline. The dose of amitriptyline was reduced to 25 mg at night as the patient complained of excessive sedation.

He remained well and maintained weekly follow-up for about 2 weeks when there was a re-appearance of low self-esteem, lack of confidence and sadness of mood, and increased anxiety with regard to attending college. He revealed that his examinations were approaching and that he was worried about performing to his earlier capacity. As he insisted that he not be given any sedating antidepressants or ECT as he was preparing for an examination, he was given risperidone 1 mg a day as an adjunct to escitalopram. He was asked to follow-up in 3 days but he failed to keep his appointment and was brought a week later by his mother with tremors, rigidity, a mask-like face, dulling of cognition and bradykinesia; problems which he said had gradually developed over 4–5 days. He insisted that he was taking the drugs at the advised doses and denied any attempt at overdose, a fact confirmed by his mother.

He was re-admitted and risperidone was withdrawn immediately. He was given trihexiphenidyl and the escitalopram was continued. Within 2 days, there was marked reduction in his complaint of dulling of cognition and a reduction in the extrapyramidal features was observed, which disappeared completely within a week. We put the patient on cognitive therapy and he successfully passed his college examinations the following week. He continues to be on escitalopram and cognitive therapy, and his depression has improved considerably.

## DISCUSSION

Escitalopram is a newer SSRI, an S-enantiomer of citalopram, which is both safe and effective in the treatment of depression. It is as effective at a dose of 10 mg as citalopram is at 40 mg.^4^ It has a faster onset of action and has almost no adverse effects when compared to citalopram.^5^ Hence, it was the antidepressant of choice in this case.

Risperidone is an atypical antipsychotic which is known to cause extrapyramidal adverse effects at higher doses.^6^ Although not an approved indication, risperidone has been used as an adjunct for the treatment of depression.^7^ We chose this drug as the patient refused the addition of any other sedating antidepressant or ECT. Risperidone is extensively metabolized by the CYP 2D6 enzyme.^8^ Escitalopram is a moderate inhibitor of the CYP 2D6 enzyme.^3^ Inhibitors of the CYP 2D6 enzyme interfere with the metabolism of risperidone leading to increased levels of the drug in the blood-stream, which might potentially lead to a higher incidence of adverse effects, especially extrapryamidal reactions.

There have been no controlled clinical trials on the interaction between escitalopram and risperidone, the former being a novel drug, introduced only recently. The present case illustrates the possibility of interactions between newer drugs and the potential dangers if one proceeds without caution. Although this interaction has been a serendipitous observation, it requires further investigation.

We acknowledge the possibility that this patient may be a poor metabolizer of substrates using the 2D6 enzyme; however, this is not such a common condition except in Caucasians.^8^ Even if this assumption is true, it only illustrates the necessity for studies in India on the CYP 450 enzyme system and various potential interactions, considering the extensive use of newer drugs in the treatment of psychiatric illnesses.
